# Voxel-based correlation of ^18^F-THK5351 accumulation and gray matter volume in the brain of cognitively normal older adults

**DOI:** 10.1186/s13550-019-0552-3

**Published:** 2019-08-23

**Authors:** Yoko Shigemoto, Daichi Sone, Miho Ota, Norihide Maikusa, Masayo Ogawa, Kyoji Okita, Harumasa Takano, Koichi Kato, Yukio Kimura, Emiko Morimoto, Fumio Suzuki, Hiroyuki Fujii, Noriko Sato, Hiroshi Matsuda

**Affiliations:** 1 Integrative Brain Imaging Center, National Center of Neurology and Psychiatry, 4-1-1, Ogawa-Higashi, Kodaira, Tokyo, 187-8551 Japan; 2 Department of Radiology, National Center of Neurology and Psychiatry, 4-1-1, Ogawa-Higashi, Kodaira, Tokyo, 187-8551 Japan; 3 Department of Clinical and Experimental Epilepsy, UCL Institute of Neurology, University College London, Gower Street, London, WC1E 6BT UK; 4 Division of Clinical Medicine, Department of Neuropsychiatry, Faculty of Medicine, University of Tsukuba, 1-1-1, Tennodai, Tsukuba, Ibaraki, 305-8576 Japan; 5 Department of Drug Dependence Research, National Institute of Mental Health, National Center of Neurology and Psychiatry, 4-1-1, Ogawa-Higashi, Kodaira, Tokyo, 187-8551 Japan

**Keywords:** Cognitively normal older adult, Medial temporal lobe, ^18^F-THK5351 PET, Gray matter volume, Primary age-related tauopathy

## Abstract

**Backgrounds:**

Although neurofibrillary tangles (NFTs) mainly accumulate in the medial temporal lobe with human aging, only a few imaging studies have investigated correlations between NFT accumulation and gray matter (GM) volume in cognitively normal older adults. Here, we investigated the correlations between ^18^F-THK5351 accumulation and GM volume at the voxel level.

**Material and methods:**

We recruited 47 amyloid-negative, cognitively normal, older adults (65.0 ± 7.9 years, 26 women), who underwent structural magnetic resonance imaging, ^11^C-Pittsburgh compound-B and ^18^F-THK5351 PET scans, and neuropsychological assessment. The magnetic resonance and ^18^F-THK5351 PET images were spatially normalized using Statistical Parametric Mapping 12. Voxel-wise correlations between ^18^F-THK5351 accumulation and GM volume were evaluated using the Biological Parametric Mapping toolbox.

**Results:**

A significant negative correlation (*p* < 0.001) between ^18^F-THK5351 accumulation and GM volume was detected in the bilateral medial temporal lobes.

**Conclusions:**

Voxel-wise correlation analysis revealed a significant negative correlation between ^18^F-THK5351 accumulation and GM volume in the medial temporal lobe in individuals without amyloid-β deposits. These results may contribute to a better understanding of the pathophysiology of primary age-related tauopathy in human aging.

## Introduction

Neuropathological studies have revealed that neurofibrillary tangles (NFTs) mainly accumulate in the medial temporal lobe (MTL) with human aging. Although neurodegeneration is also a feature of aging, in addition to tau pathology, only a few imaging studies have specifically investigated correlations between tau accumulation and brain volume [1, 2]. Although ^18^F-THK5351 was originally developed as a tau-specific tracer, recent studies have clarified the off-target binding to monoamine oxidase B (MAO-B) [3]. Because ^18^F-THK5351 accumulation reflects the combination of astrogliosis, in addition to tau pathology, it is now considered the promising biomarker for detecting neuroinflammation [4]. Previously, using region of interest (ROI) analysis, we did not detect significant negative correlations between ^18^F-THK5351 accumulation and gray matter (GM) volume [5]. In this study, we aimed to re-examine the correlations between ^18^F-THK5351 accumulation and GM volume at the voxel level.

## Materials and methods

### Participants

We recruited 47 cognitively normal older adults from the Brain Mapping by Integrated Neurotechnologies for Disease Studies (Brain/MINDS) project (grant number 18dm0207017h0005), who underwent structural magnetic resonance imaging and ^11^C-Pittsburgh compound-B (PiB) and ^18^F-THK5351 PET scans. All participants underwent cognitive testing that included the Mini-Mental State Examination (MMSE), global Clinical Dementia Rating Scale (CDR), and Wechsler Memory Scale-Revised Logical Memory II (WMSR LM-II). The inclusion criteria were the following: visually negative PiB PET results, a global CDR of 0, an MMSE ≥ 26, and performance within education-adjusted norms for WMSR LM-II, absence of neurological or psychiatric disorders, and no medications that affect cognition.

### Data acquisition

All participants underwent 3D T1-weighted scans with a 3-T magnetic resonance imaging system (Verio; Siemens, Erlangen, Germany). PET scans were acquired using a Siemens/Biograph TruePoint16 Scanner (3D acquisition mode; 81 image planes; 16.2-cm axial field of view; 4.2-mm transaxial resolution; 4.7-mm axial resolution; 2-mm slice interval). For ^11^C-PiB imaging, participants were injected with 555 ± 185 MBq of PiB prior to imaging and imaging was performed for a 20-min PET acquisition, 50 ± 5 min post-injection. For ^18^F-THK5351 imaging, participants were injected with 185 ± 37 MBq of THK5351 prior to imaging and imaging was performed for a 20-min PET acquisition, 40 ± 5 min post-injection. PET/CT data were reconstructed using an iterative 3D ordered subset expectation maximization reconstruction algorithm.

### Data preprocessing

The partial volume corrected ^18^F-THK5351 PET images using PETPVE12 toolbox [6] were normalized using SPM12 (Statistical Parametric Mapping 12; Wellcome Department of Cognitive Neurology, London, England). Each participant’s ^18^F-THK5351 PET image was coregistered to its T1-weighted image and normalized with Diffeomorphic Anatomical Registration Through Exponentiated Lie Algebra. A transformation matrix was applied to each ^18^F-THK5351 PET image, which had been coregistered to the T1-weighted images. After spatial normalization, standardized uptake value ratios (SUVRs) for ^18^F-THK5351 PET images were calculated using the individual’s positive mean uptake value of cerebellar GM as the reference region. Finally, each PET image was smoothed by an 8-mm full width at half maximum (FWHM) Gaussian kernel. The GM images segmented using SPM12 were also normalized and smoothed using an 8-mm FWHM Gaussian kernel.

### Statistical analyses

The Biological Parametric Mapping (BPM) toolbox allows voxel-level comparisons across imaging modalities based on the general linear model to perform regressions [7]. Using the BPM toolbox, we evaluated the direct correlations between ^18^F-THK5351 accumulation and GM volume at the voxel level. Results were considered significant at *p* < 0.001 with an extent threshold of 30 voxels (uncorrected for multiple comparisons).

## Results

The participants’ demographics are shown in Table 1. Mean age ± standard deviation was 65.0 ± 7.9 years and mean cognitive scores were 29.3 ± 1.1 for MMSE and 13.4 ± 2.9 for WMSR LM-II.

**Table 1:** Participants’ demographics

	Cognitively normal older adults
No. (% women)	47 (26)
Age, year	65.0 ± 7.9 [50–86]
Education, year	14.3 ± 2.4 [9–22]
MMSE	29.3 ± 1.1 [26–30]
WMSR LM-II	13.4 ± 2.9 [8–19]

Values are mean ± standard deviation [range]. *MMSE* Mini-Mental State Examination, *WMSR LM-II* Wechsler Memory Scale-Revised Logical Memory II

**Fig. 1:**
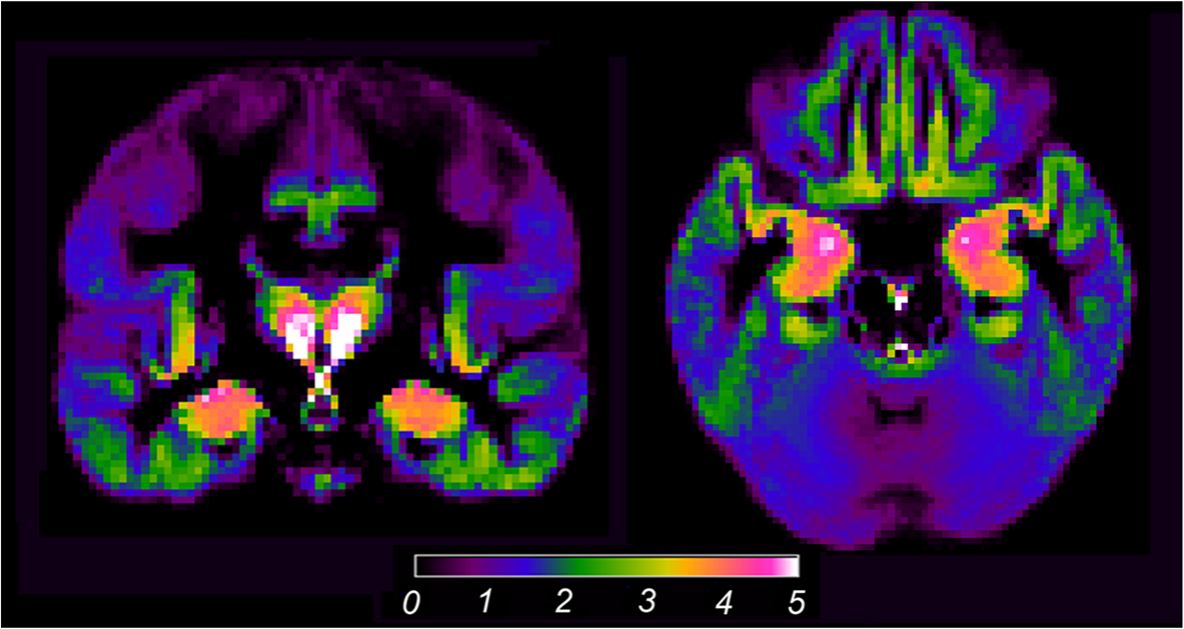
Mean SUVR images of ^18^F-THK5351 in cognitively normal older adults. Localized ^18^F-THK5351 accumulation was mainly evident in the basal ganglia, thalamus, and medial temporal lobe, which slightly extended to the inferior temporal lobe, insula, posterior cingulate gyrus/precuneus, and basal frontal lobe. SUVR, standardized uptake value ratio

**Fig. 2:**
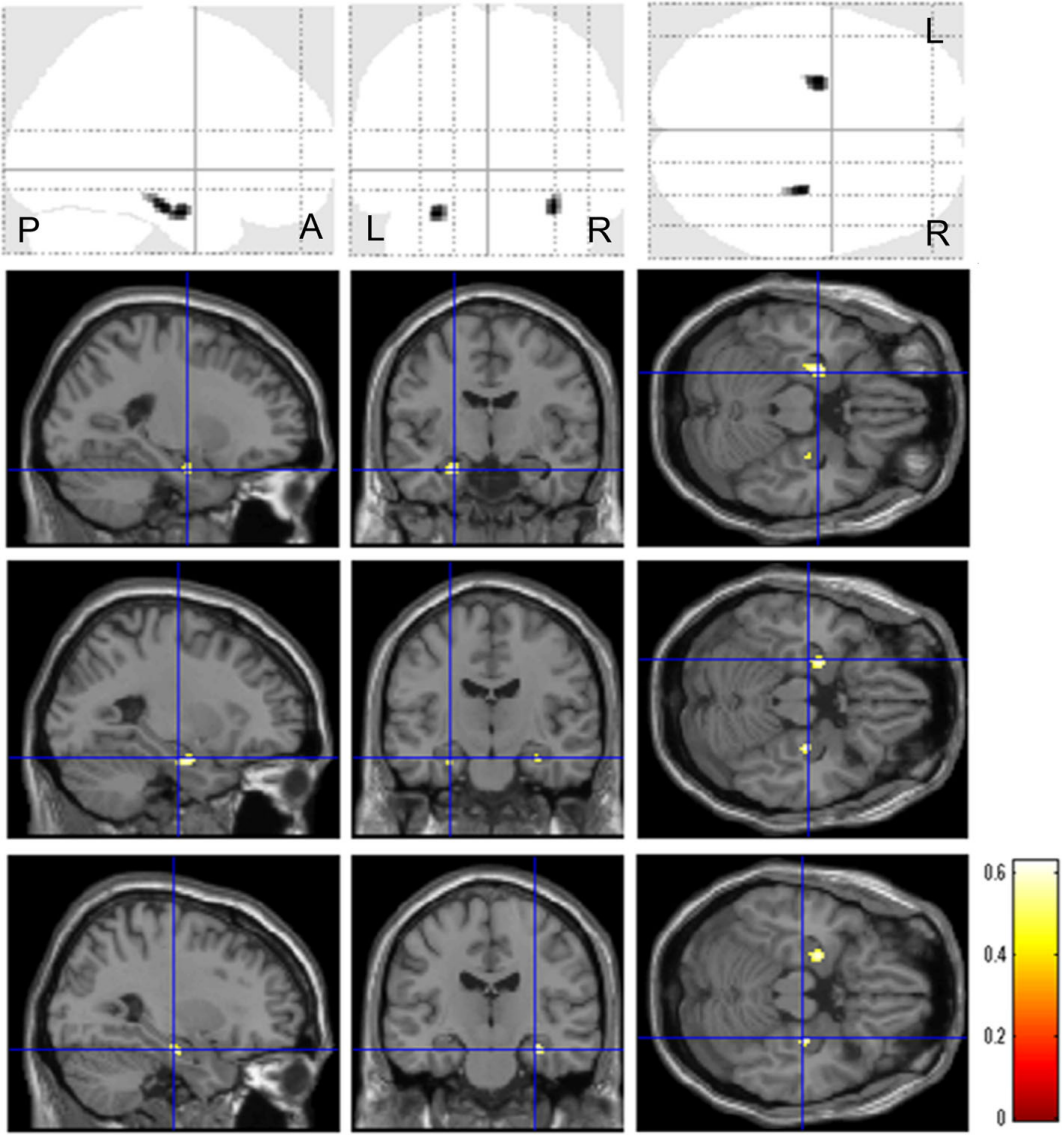
Voxel-wise correlations between ^18^F-THK5351 accumulation and gray matter volume in normal older adults. Significant negative correlations between ^18^F-THK5351 accumulation and gray matter volume were detected in the bilateral medial temporal lobes (voxel threshold of *p* < 0.001 with a 30-voxel extent threshold). A anterior, P posterior, R right, L left

Localized ^18^F-THK5351 accumulation was detected mainly in the basal ganglia, thalamus, MTL but slightly extended into the inferior temporal lobe, insula, posterior cingulate cortex/precuneus, and basal frontal lobe (Fig. 1). We found a significant negative correlation between ^18^F-THK5351 accumulation and GM volume in the bilateral MTL, right parahippocampal gyrus (cluster size, 40 voxels; *Z*-score, 4.68; MNI coordinate [*x*, *y*, *z*], [32, − 18, − 18]), right hippocampus, left parahippocampal gyrus (cluster size, 56 voxels; *Z*-score, 4.68; MNI coordinate [*x*, *y*, *z*], [− 26, − 8, − 22]), and left amygdala (Fig. 2).

## Discussion

This is the first study to investigate direct correlations between ^18^F-THK5351 accumulation and GM volume at the voxel level using BPM in cognitively normal older adults. We detected significant correlations between an increased ^18^F-THK5351 accumulation and reduced GM volume in the MTL. Our findings may contribute to a better understanding of the pathophysiology of human aging.

We found significant ^18^F-THK5351 accumulation mainly in the basal ganglia, thalamus, MTL slightly extending into the inferior temporal lobe, insula, posterior cingulate cortex/precuneus, and basal frontal lobe. Our finding corresponded to Braak stage III–IV, which is not consistent with previous neuropathological and tau PET studies that tau pathology was usually localized in the MTL in the cognitively healthy participants [8, 9]. However, a recent large cohort study [10] showed elevated ^18^F-THK5351 tau tracer retentions in Braak stage III–IV areas with normal amyloid status and raised the possibility of primary age-related tauopathy (PART) [11]. Our results also support their results and might reflect PART. PART is defined as NFT pathology mostly restricted to the MTL, basal forebrain, brainstem, and olfactory areas in the absence of β-amyloid in the aged brain. Cognitive function is usually normal to mildly impaired in PART, and severe cognitive decline is rarely seen. Although PART is suggested to be an age-related phenomenon distinct from early Alzheimer’s disease, its pathophysiology is still unclear [1]. Thus, further studies with tau PET are needed to better understand the pathogenesis of PART.

The BPM analysis demonstrated a significant voxel-wise negative correlation between ^18^F-THK5351 accumulation and GM volume in the MTL, which was not detected in the previous ROI-based analysis [5]. Although ROI analysis is a common approach, it may not be able to accurately assess localized accumulation due to dilution effects. Because the BPM toolbox enables a direct comparison across imaging modalities at the voxel level, this voxel-based analysis may be more reliable than traditional ROI-based analyses. Our findings are consistent with neuropathological studies of PART patients showing that a higher Braak NFT stage is associated with hippocampal head atrophy [1]. Similar findings have been reported in recent ^18^F-AV1451 tau PET studies, namely, that higher MTL tau is associated with MTL atrophy [2]. Although they managed to obtain these results using a predefined FreeSurfer ROI approach, it is possible that nonspecific ^18^F-AV1451 accumulation in the choroid plexus adjacent to the MTL may not have been eliminated.

This study has several limitations. First, the number of participants was relatively small. Second, the study lacked pathologic confirmation of tau pathology. Third, the high affinity of ^18^F-THK5351 to MAO-B [3, 4] may contribute to the relatively higher Braak stage of our findings compared to PART type pathology which is usually III or lower [11]. MAO-B concentration increases during ongoing astrogliosis, which is considered as neuroinflammation changes that occur in response to brain injury and neurodegenerative disease [12]. Previous PET study in healthy subjects reported global MAO-B increases in the whole brain even with human aging [13], and the astrocytes seem to contribute to low-grade inflammation in the aged brain [14]. Because ^18^F-THK5351 uptake reflects the combination of astrogliosis and tau pathology, the degree and extent of tracer retention could be higher than that of only tau pathology. Recently, it is reported that aggregation of misfolded proteins including TDP-43 and α-synuclein in addition to Aβ and NFTs observed in PART are common even in cognitively healthy elderly brain [15]. Since the mixed pathologies are frequently observed in the aged brain, they could evoke neuroinflammation and increase astrogliosis, resulting in accumulation of ^18^F-THK5351. The early phase of age-related TDP-43 accumulation, known as “limbic-predominant age-related TDP-43 encephalopathy (LATE),” which tends to extend to limbic areas including the amygdala, could be part of the reasons [16]. In addition, the stereological cell counting studies showed declining of neocortical neuronal populations but no changes of the total astrocyte numbers in the aged human brains [17]. Therefore, the numbers of astrocytes would tend to concentrate in the atrophied MTL regions, suggesting the contribution of MAO-B in addition to tau pathology.

In summary, we found significant voxel-wise negative correlations between ^18^F-THK5351 accumulation and GM volume in the MTL. These results may reflect the concept of PART and contribute to a better understanding of the neurobiology of aging. Further studies are needed to confirm whether our findings reflect PART pathology or not by taking an oral dose of MAO-B inhibitor selegiline [3] or using second-generation tau-specific tracers with much less off-target binding.

## Data Availability

The datasets used and analyzed during the current study are available from the corresponding author on reasonable request.

## Abbreviations

BPM: Biological Parametric Mapping
CDR: Clinical Dementia Rating
FWHM: Full width at half maximum
GM: Gray matter
MAO-B: Monoamine oxidase B
MMSE: Mini-Mental State Examination
MTL: Medial temporal lobe
NFT: Neurofibrillary tangle
PART: Primary age-related tauopathy
PiB: Pittsburgh compound-B
ROI: Region of interest
SPM: Statistical Parametric Mapping
SUVR: Standardized uptake value ratio
WMSR LM-II: Wechsler Memory Scale-Revised Logical Memory II
